# Weak Structure and Environment‐Associated Loci Across a Eutrophication Gradient in a Resilient Coral Species

**DOI:** 10.1002/ece3.73908

**Published:** 2026-07-01

**Authors:** Le Qin Choo, Vriko Yu, Paolo Momigliano, Shelby E. McIlroy

**Affiliations:** ^1^ Simon F.S. Li Marine Science Laboratory, School of Life Sciences The Chinese University of Hong Kong Hong Kong China; ^2^ School of Biological Sciences, The Swire Institute of Marine Science The University of Hong Kong Hong Kong China; ^3^ Department of Ecological and Biological Sciences University of Tuscia Viterbo Italy

**Keywords:** adaptation, genome‐wide, *Oulastrea crispata*, population structure, RADseq

## Abstract

In marine species, large effective population sizes and high connectivity minimise drift and maintain genetic diversity. However, it is uncertain how this influences their ability to adapt to projected environmental changes from anthropogenic effects. Scleractinian corals are keystone species that face increasing threats from coastal pollution and climate change. Here we used RADseq to examine the population structure of *Oulastrea crispata,* an encrusting coral native to the Indo‐Pacific that inhabits a large geographic range and exhibits high tolerance to environmental stress. To assess potential adaptive differences in *O. crispata* , we sampled individuals from across a spatially small but pronounced eutrophication gradient (Dissolved inorganic nitrogen: 1.6–9.0 μM) within Hong Kong. A set of 39,527 genome‐wide single nucleotide polymorphisms (SNPs) revealed no broad neutral population structure within our dataset of 90 individuals despite variation in environmental conditions. The level of diversity estimates and low inbreeding coefficient suggest no loss of diversity in coral population despite the challenging environment. While *F*_ST_ outlier analyses failed to reveal any clear signature of selection across sites, genotype‐environment association analyses identified 137 outlier SNPs associated with several water quality parameters. The transcriptome‐based annotation of these outlier loci indicates that their potential roles in environmental response. We also find that across the eutrophication gradient we sampled, *O. crispata* was consistently associated with the environmental stress‐tolerant symbiont genus *Durusdinium* sp. The presence of environment‐associated alleles, despite panmixia at nuclear loci, is compatible with adaptive responses driven by local environmental conditions. The detection of a stress‐tolerant Symbiodiniaceae species suggests a complementary holobiont‐level mechanism. Phenotypic plasticity of *O. crispata* could be another mechanism for stress resilience. Future additional studies using whole genome sequencing and transcriptome analyses will be needed to clarify the genetic architecture of host resilience in *O. crispata* and the relative contributions from host genetics and holobiont‐mediated effects.

## Graphical abstract

Marine species often have large effective population sizes and high connectivity that reduce drift and preserve diversity, but how these features shape adaptation to anthropogenic change remains unclear; *Oulastrea crispata* , a stress‐tolerant Indo‐Pacific coral, is an ideal test case along a strong eutrophication gradient. Using RADseq on 90 individuals across a dissolved inorganic nitrogen gradient (1.6–9.0 μM) in Hong Kong, we found no population structure and mixed evidence for genotype‐environment associations, with healthy diversity, low inbreeding and environment‐associated loci. These results are compatible with adaptive responses driven by local environment conditions and guide future work towards integrative studies of genetic architecture, plasticity, and microbiome‐mediated symbioses under coastal pollution and climate change.

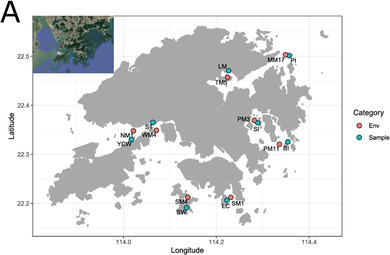

## 1 Introduction

How populations evolve depends on the interplay between selection and gene flow. Local adaptation depends on gene flow to increase genetic variation, which allows beneficial alleles to spread spatially and persist across generations (Tigano and Friesen 2016). Gene flow can also have a negative effect on local adaptation: when migration is stronger than selection local adaptation is prevented in a process known as gene swamping (Kawecki and Ebert 2004). In marine habitats, the potential for gene flow can be high, especially for organisms with pelagic life stages, as coastal and oceanic currents are the main drivers of their dispersal (e.g., corals: Feng et al. 2016; mussels: Mackenzie et al. 2022; clownfish: Simpson et al. 2014). How these processes interact across time and space can differ across species, nevertheless, for organisms undergoing strong selective pressures it is likely that local adaptation will proceed despite high gene flow (López‐Goldar and Agrawal 2021). In systems with high connectivity, local adaptation is generally expected to result from large effect loci or tightly linked clusters of small‐effect loci that act as a single unit as only such architecture can withstand the homogenising effects of gene flow and prevent gene swamping (Yeaman and Whitlock 2011).

Scleractinian corals are currently facing strong selective pressures due to climate change and other anthropogenic impacts. These stressors include temperature extremes, ocean acidification, salinity changes and coastal eutrophication (Helgoe et al. 2024). These disturbances act synergistically, increasing the pressure on coral survival (Setter et al. 2022). With 997 million people living within 100 km of coastal coral populations, coral reef ecosystems provide vital ecosystem services to human settlements. Despite this, coral ecosystems are urgently threatened by the effects of coastal urbanisation such as pollution, habitat destruction, and overfishing (Heery et al. 2018). Studies have shown that corals have the capacity to adapt to environmental gradients, for example, in *Acropora palmata* in the Caribbean (Devlin‐Durante and Baums 2017), *A. millepora* on the Great Barrier Reef (though at scales of ~700 km) (Matz et al. 2018) and *Seriatopora hystrix* in the Red Sea and the Indian Ocean (van der Ven et al. 2021), with environmental gradients or habitat discontinuities found to drive genetically distinct subpopulations within single reef systems. However, to fully understand how species cope with immediate and projected stressors at local scales, it is essential to study not only coral adaptation to climate change (Bay and Palumbi 2014; Bairos‐Novak et al. 2021) but also how they maintain resilience in increasingly urbanised habitats, especially with the rapid timescales of anthropogenic change.

Marginal coral reef habitats, which exist in suboptimal environmental conditions (Perry and Larcombe 2003), are a valuable natural laboratory for investigating the effects of combined stressors on coral survival (van Hooidonk et al. 2014). Urbanisation, eutrophication, and natural estuarine influences affect water quality metrics in Hong Kong with pronounced gradients across tens of kilometres. Western waters experience raised dissolved inorganic nitrogen levels and turbidity and fluctuating salinity due to run‐off from anthropogenic sources and the Pearl River Delta, while eastern waters remain relatively oligotrophic with constant oceanic input (Duprey et al. 2016). These local differences in dissolved nitrogen, salinity and turbidity may impose divergent selective pressures across short distances, allowing us to study the effect of gene flow and local adaptation to urbanised, disturbed habitats. Hence, coral habitats in Hong Kong offer a valuable setting for studying adaptation and resilience under environmental stress (Duprey et al. 2017).

Genetic adaptation to varied environmental stressors can be mediated through either small effect loci dispersed across the whole genome or through large effect and/or closely linked loci. Physiological changes that improve fitness and survival in coral through fluctuating thermal conditions can be attributed to many loci of small effect (Bay and Palumbi 2014). In contrast to adaptation being driven by small effect loci, it has been observed that when gene flow is high among coral populations experiencing spatially variable heat stress, the loci under selection tend to be tightly clustered and exhibit high linkage disequilibrium (Thomas et al. 2022). In either instance, these genomic signatures are more likely to be identified through genome‐wide methods that enable the querying of thousands of sites across the genome to identify sites that show evidence of selection (i.e., GWAS after whole genome resequencing) and linkage disequilibrium. Many marine organisms still lack genomic resources, especially if they have low commercial value or are not model organisms. Hence reduced representation approaches, particularly RADseq‐based approaches like RAD‐Tag (Baird et al. 2008), ddRAD‐seq (Peterson et al. 2012) and 2b‐RAD (Wang et al. 2012) are useful to query genome‐wide information with no prior genomic information and can capitalise on existing software to detect selection and gene flow with such datasets.

To examine the role of adaptation in eutrophication resistance, we focused on the environmentally tolerant zebra coral *Oulastrea crispata* that inhabits waters with a broad range of dissolved nitrogen contents. *O. crispata* ranges latitudinally from the tropical Indo‐Pacific to temperate zones such as Jeju Island and southern Japan and has been recorded as an introduced species in the Mediterranean (Chen et al. 2003; Hoeksema and Ocana Vicente 2014; Mariani et al. 2018; Park et al. 2019). In Hong Kong, it dominates turbid intertidal and sub‐tidal zones, seawalls, and shaded crevices (Lam 2000), including regions where few other coral species persist (Duprey et al. 2016). Beyond its environmental tolerance, *O. crispata* is reproductively versatile as a hermaphroditic coral capable of both sexual (broadcast spawning and brooding) and asexual reproduction (intra‐ and extra‐tentacular budding) and is also known for its trophic flexibility (being able to switch from being partially reliant of endosymbionts for nutrients to being entirely heterotrophic) (Chei et al. 2025). These life history traits, combined with environmental tolerance, underscore its potential as a model organism for exploring coral resilience under marginal conditions (Röthig et al. 2020). Hence, we can study its genetic response to eutrophication using RADseq, which enables us to study genome‐wide signals without the need for prior genomic resources. We hypothesised that *O. crispata* 's success across a strong environmental gradient in Hong Kong reflects local adaptation to site specific stressors, including pollution and other anthropogenic impacts.

## 2 Materials and Methods

### 2.1 Environmental Data and Site Selection

**FIGURE 1:**
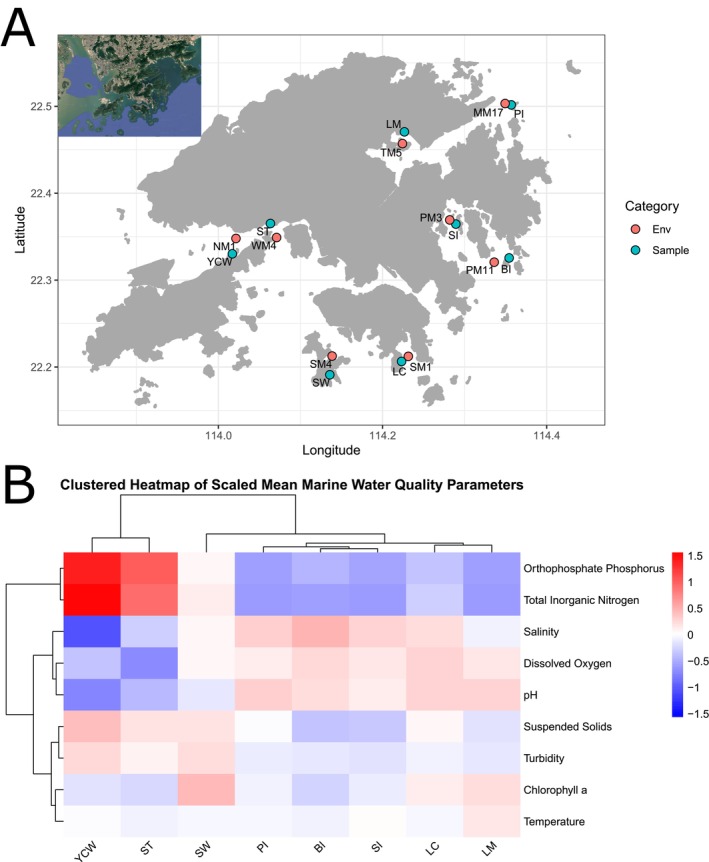
Sampling locations of *Oulastrea crispata* and their environmental parameters across Hong Kong. (A) Figure shows the eight sites (Sample) sampled in August 2018 with site abbreviations corresponding to Table 1 and the corresponding environmental sampling stations (Env) by the EPD. The inset map (top left) shows the position of Hong Kong relative to the Pearl River Delta. Satellite image obtained from Google Earth. (B) Clustered heatmap of the scaled mean water quality parameters for each environmental monitoring station (labelled as nearest sampling station), positive values indicate higher values relative to the global mean across the sites, while negative values are reduced relative to the mean across sites.

Sampling sites were selected along the West–East water quality gradient across Hong Kong (Table 1 and Figure 1A). Waters in the west are more eutrophic (having higher values of chlorophyll *a*, dissolved inorganic nitrogen, phosphate, oxygen and particulate matter while having lower salinity) and are linked with lower coral cover and species richness, while the Eastern waters are influenced by oceanic water mixing and support higher coral diversity (Duprey et al. 2016).

**TABLE 1:** Details of sample collection and water quality categories based on environmental variables from the EPD.

Sample collection location	Number of samples collected per location	GPS coordinates	Collection date	EPD monito‐ring station	Water quality
Sham Wan (SW)	6	22.191094, 114.135742	8/8/2018	SM4	Med
Lo Chau (LC)	12	22.206406, 114.223178	8/8/2018	SM1	High
Bluff Island (BI)	15	22.32555, 114.354244	9/8/2018	PM11	High
Sharp Island (SI)	4	22.364486, 114.289325	9/8/2018	PM3	High
Lung Mei (LM)	18	22.470633, 114.226647	14/8/2018	TM5	High
Port Island (PI)	6	22.501664, 114.356817	14/8/2018	MM17	High
Sam Tseng (ST)	12	22.365128, 114.063428	16/8/2018	WM4	Low
Yam Chai Wan (YCW)	17	22.330284, 114.017023	16/8/2018	NM1	Low

For this study, water quality data were obtained from the HKSAR Environmental Protection Department (EPD) which conducts monthly monitoring at several stations across Hong Kong. For each sampling site, we used measurements from the nearest EPD environmental monitoring station as a proxy for the site's environmental conditions (Figure 1A). Surface water parameters (measured 1 m below the surface) were used, as scleractinian corals in Hong Kong are found in shallow waters between 2 and 5 m (Morton 1994). This study included nine water quality parameters previously correlated with coral survival in Hong Kong (Duprey et al. 2016; Cybulski et al. 2020) and processed such that values below detection limits were replaced with values half of the detection limit.

To identify similarities among sites based on their environmental parameters, we averaged and scaled to z‐scores the monthly measurements of the variables collected from August 2013 to July 2018 (a total of 60 months leading up to the time of sampling) for each of the eight environmental monitoring stations. We then created a heatmap using the pheatmap package (Kolde 2025) in R, applying Euclidean distances and Ward's D2 clustering method.

### 2.2 Sample Collection

A total of 90 individuals of *Oulastrea crispata* were collected across the selected sites in August 2018, with 4–17 samples obtained per site (Table 1 for detailed coordinates and sample counts). Coral fragments were removed using a hammer and chisel during SCUBA dives. Immediately after collection, samples were brought to the surface, rinsed with deionised water, wrapped in aluminium foil, and frozen in liquid nitrogen to preserve DNA quality.

### 2.3 DNA Extraction and Sequencing

Genomic DNA was extracted from all samples using the DNeasy Blood & Tissue Kit (Qiagen, Valencia, CA, USA) according to the protocol provided by the manufacturer. DNA extracts were sent to Floragenex Inc. (Beaverton, OR, USA) for RADseq library construction with the PstI enzyme (Baird et al. 2008). Single‐end 122 bp sequencing was performed on the NovaSeq 6000 at the University of Oregon GC3F facility in Eugene, OR, USA to obtain a minimum of 1 M reads per individual.

### 2.4 Read Analysis

The pipeline for RADseq assembly and population genomics analyses is illustrated in Figure S1 and described in subsequent sections. Firstly, the sequencing data were processed to generate loci using the de novo Stacks pipeline v2.68 (Rochette et al. 2019). The raw FASTQ reads were first cleaned using the process_radtags function and then assembled into putative alleles using ustacks and passed to cstacks to construct a catalogue of consensus loci by merging alleles. All samples were subsequently mapped to the generated catalogue of loci using sstacks, and the resulting data were processed into BAM files using tsv2bam. These files were then aligned by locus using gstacks with the default Marukilow SNP model to discover SNPs and call genotypes, with an alpha threshold of 0.05 for both SNP discovery and genotype calling. The final dataset was passed to the populations module, which computed population‐level statistics and produced a VCF file for further analysis.

Several parameter configurations for ustacks and cstacks were explored to identify the optimal settings for (i) m (minimum required read coverage depth to form a stack, tested in the range of 3–7), (ii) M (maximum number of mismatches allowed between stacks to consider them as the same locus, tested in the range of 1–8), and (iii) n (maximum number of mismatches allowed between loci from different individuals to be considered homologues, tested in the range of 7–9). The aim was to maximise the number of polymorphic loci shared by at least 80% of individuals (Paris et al. 2017). Based on this criterion, the optimal parameter settings were determined to be *m* = 3, *M* = 8, and *n* = 9. This configuration resulted in a mean sequencing depth per individual of 37.8× (SD = 23.4×), with depths ranging from 9× to 160.5×.

### 2.5 Removal of Contaminant Loci and Read Mapping to Symbiodiniaceae

Taxonomic classification of loci was performed using Kraken2 (Wood et al. 2019) to remove potential contaminants from non‐coral sources. The catalogue of loci generated by cstacks was compared against the RefSeq Standard, Protozoa & Fungi database (https://benlangmead.github.io/aws‐indexes/k2), as well as a database of assembled genomes from the class Dinophyceae (Table S1). Contamination rates were identified as 3.87% matching PlusPF sequences and 10.9% matching Dinophyceae sequences. These contaminant loci were removed from the VCF file, resulting in a cleaned VCF file with 5,213,563 SNPs (85.2%) likely derived from *Oulastrea crispata* .

To explore the association of *Oulastrea crispata* with the various endosymbiont strains, RADseq reads from individuals were mapped to 11 Symbiodiniaceae references within the Dinophyceae set (Table S1) with bbmap version 39.81 (Bushnell et al. 2017) using default settings. The number of unambiguously mapped reads to each genome (i.e., uniquely mapping to only one genome) (Data S1) was transformed without normalisation into a Bray–Curtis dissimilarity matrix using the vegdist function and compared across water quality categories using PERMANOVA with the adonis2 function from the vegan package (Oksanen et al. 2022). No zeros were present within the mapping results; hence no adjustments were made. Statistical significance was evaluated after 999 permutations. The homogeneity of dispersion among water quality groups (betadisper) not significant (*F* = 2.207, *p* = 0.114), hence the assumption of equal group variance is satisfied for PERMANOVA. The percentage of all reads that were mapped to the each Symbiodiniaceae reference, before and after log 10 transformation, were plotted.

### 2.6 Population Structure Analyses

The VCF file was filtered using BCFtools (Danecek et al. 2021) to retain only biallelic SNPs with a minimum depth of 5 and missingness below 20% (Filtered VCF, 39,527 loci). To reduce the impact of linkage disequilibrium, we selected one random SNP per locus, resulting in an LD pruned dataset of 39,527 unlinked SNPs. To estimate relatedness among all individuals in the dataset, fineRADstructure (Malinsky et al. 2018) was conducted on a subset of 81 individuals with less than 20% missingness across the filtered VCF that were not filtered for linkage disequilibrium. Two pairs with high relatedness were identified (Figure S1), and one of each pair was removed in downstream to remove the bias from having related individuals for population genetic analyses. Principal component analysis (PCA) was conducted on the LD pruned VCF using the adegenet package (Jombart 2008; Jombart and Ahmed 2011) in R4.3.1 (R Core Team 2023) to visualise genetic relationships across sampling locations for the remaining 88 individuals. An ADMIXTURE v1.3.0 (Alexander et al. 2009) analysis was also performed, testing values of *K* (number of ancestral populations) from 1 to 5 (Table S2).

### 2.7 Population Diversity Estimates

Population diversity metrics, including observed heterozygosity, expected heterozygosity, nucleotide diversity (π), and inbreeding coefficient (Fis), were calculated within each sampling location using the population module in Stacks v2.68. These calculations were performed across both variant and fixed sites, including loci with a minimum depth of 3, present in at least one sampling location, and present in at least 25% of individuals within a given location (Table S3). For the following population structure analyses, a minor allele frequency (MAF) threshold of 0.05 was applied to the LD pruned dataset, leading to a MAF filtered set of 20, 731 SNPs. A hierarchical Analysis of Molecular Variance (AMOVA) was calculated with the R package StAMPP (Pembleton et al. 2013). The distribution of variation between sampling locations was calculated with pairwise Weir and Cockerham's (1984) Fst using the function pairwise.WCfst and tested for significant deviations from zero (100 bootstraps, 0.95 confidence interval) with boot.ppfst from the R package hierfstat (Goudet and Jombart 2024).

### 2.8 Outlier Analyses

Outlier analyses were conducted on the LD pruned dataset with BayeScan v2.1 (Foll and Gaggiotti 2008) and OutFLANK v0.2 (Whitlock and Lotterhos 2015), while genotype‐environment associations of these SNPs were conducted with processed environmental data (see methods section: 2.1 Environmental data and Site Selection). A univariate approach was conducted using (LFMM) Latent Factors Mixed Models in R (Caye et al. 2019) and redundancy analysis (RDA) as implemented in vegan (Oksanen et al. 2022) was chosen as the multivariate approach. Parameters for implementation are described in Table 2, with the response variable as genotypes for all these tests. For the BayeScan and OutFLANK analyses, individuals were pooled into water quality categories and analysed across a single simultaneous run. In the LFMM analysis, we used the lfmm ridge approach with predictor variable as the PC1 of the scaled environmental data and the number of latent factors chosen was *K* = 1, as it best described the neutral population structure present within the dataset. To reduce the effect of false positives by uneven sampling sizes, we identify outliers only if they have been identified by more than one of these methods. For RDA, the collinear environmental parameters were removed by variance inflation factor analysis (VIF analysis), where the predictor with highest VIF was removed after fitting of the RDA and computation of VIF predictor, if above the threshold of 10. This process was repeated until all variables had VIF below the threshold. This resulted in a final set of five environmental variables: Chlorophyll *a* (CHL), Dissolved oxygen (DO), pH, Suspended Solids (SS) and Temperature (TEMP) for RDA. No conditioning variables were used. Permutation tests were conducted to check the significance of the overall model and each constrained axes, while outlier SNPs were tested for significance with Mahalanobis distances estimated between each locus and the centre of the RDA, partialling out the first two axes of the PCA to account for structure. The Mahalanobis distances were corrected for the inflation factor and transformed into *p*‐values using a chi‐squared distribution with two degrees of freedom as in (Capblancq et al. 2018).

**TABLE 2:** Implementation details for the outlier/GEA tests conducted.

Method	Predictors	Grouping	Parameters
Bayescan	Water quality grouping	Water quality: Low (YCW, ST) = 27 Med (SW) = 6 High (Remaining sites) = 55	‐n 5000 ‐burn 50,000 ‐pr_odds 1000 Outlier detection threshold *Q* = 0.05
OutFLANK	Water quality grouping		Default settings: LeftTrimFraction = 0.05, RightTrimFraction = 0.05, Hmin = 0.1, qthreshold = 0.05
LFMM	PC1 of environmental variables	No groupings	Genetic PCA = no population structure No. of latent factors (*k*) = 1 Q threshold = 0.05
RDA	Five Environmental variables (after VIF filtering) within each site	No groupings	VIF threshold = 10 Anova number of permutations =999 Anova number of permutations for axes = 999

To functionally annotate the outlier loci identified using RDA, we mapped them using blastn at default settings to the *Oulastrea crispata* transcriptome (Quek and Huang 2019). This resulted in 95 matched transcripts, which were then annotated with Blast2Go using default settings of blastx‐fast, nr sequence database and e‐value of 1.0E‐3. The protein blast hits were mapped to the gene ontology annotated proteins (Goa version 2025.03; Gotz et al. 2008) and annotated with default settings (Annotation cut‐off: 55, GO Weight: 5, E‐value‐Hit‐filter: 1.0E‐6, Hit filter: 500). InterProScan was merged with GO terms to improve annotation.

## 3 Results

### 3.1 Homogeneous Population Structure With Subtle Localised Divergence

**FIGURE 2:**
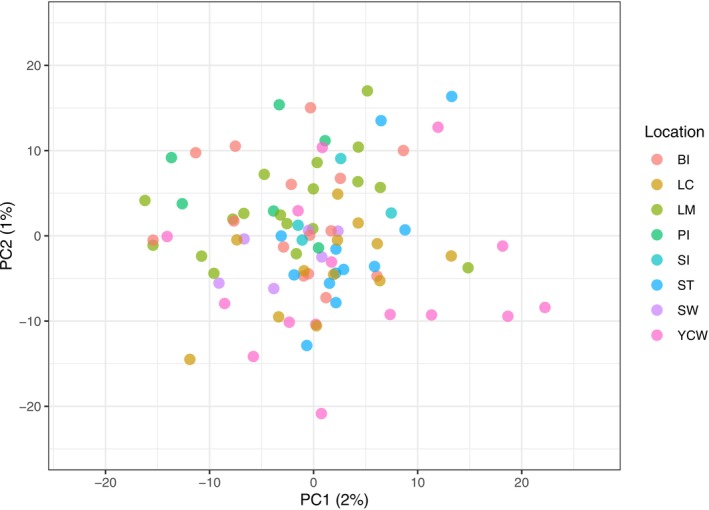
Principal component analysis of 88 *Oulastrea crispata* individuals, after removing two related individuals based on 39,527 unlinked SNPs.

*Oulastrea crispata* individuals sampled across Hong Kong revealed a genetically homogeneous population. After the removal of two individuals with high relatedness to other individuals in the dataset (Figures S2 and S3), the principal component analysis (PCA) based on 39,527 unlinked SNPs and 88 remaining individuals shows no clear genetic structure, with the first two principal components (PC1 and PC2) each explaining only 2% and 1% of the total variation respectively (Figure 2). ADMIXTURE analysis further supports this genetic uniformity. The optimal value of K = 1, identified by the lowest cross‐validation error for the various K values sampled (CV error = 0.55) (Figure S4 and Table S2).

### 3.2 Population Diversity Estimates

Minimal differences in heterozygosity and nucleotide diversity were observed among sampling locations. Observed heterozygosity (Obs. Het., range: 0.00589–0.00664) was consistently lower than expected heterozygosity (Exp. Het., range: 0.00865–0.0134) within sampling locations, while nucleotide diversity (π) values spanned from 0.01095 in the location with fewest individuals (SI = 4) to 0.01417 in the location with largest sample size (LM = 18) (Table 3). AMOVA indicated that most of the genetic variation within the dataset was found within individuals (71.16%, *p*‐value = 0.01), followed by between individuals within populations (28.76%, *p*‐value = 0.01), with between populations comparisons accounting for only 0.08% of total variation (*p*‐value = 0.14). The inbreeding coefficient (Fis) estimates were similarly consistent across locations, with mean values ranging from 0.01342 (PI) to 0.02405 (LM), indicating limited evidence of inbreeding within sampling locations. Variance estimates for these diversity metrics were generally comparable, although slightly higher in sampling locations with smaller sample sizes. RADseq datasets may be affected by method specific biases, such as restriction‐site polymorphism and locus dropout, so results may not be directly comparable across distinct methods. Yet, the relative comparisons between sampling locations indicate a homogeneous population structure within this dataset.

**TABLE 3:** Summary of genetic diversity metrics for *Oulastrea crispata* populations across sampling locations in Hong Kong calculated from all variant and invariant sites that are present in more than 20% of individuals. Values include observed heterozygosity (Obs. Het.), expected heterozygosity (Exp. Het.), nucleotide diversity (π), and inbreeding coefficient (Fis), calculated from variant and fixed positions.

Pop ID	Num_Indv	Obs. het.	Obs. het. variance	Exp. het.	Exp. het. variance	π	π variance	Fis	Fis variance
YCW	15	0.00624	0.00171	0.0130	0.00447	0.01381	0.00507	0.02101	0.01821
ST	12	0.00589	0.00171	0.0125	0.00445	0.01364	0.0053	0.01942	0.01727
PI	6	0.00664	0.00259	0.0114	0.00437	0.01326	0.00593	0.01342	0.01313
LM	18	0.00589	0.00144	0.0134	0.00455	0.01417	0.00508	0.02405	0.02039
SW	6	0.00592	0.00233	0.0110	0.00426	0.01291	0.00601	0.01372	0.01323
BI	15	0.00616	0.00163	0.0132	0.00452	0.01404	0.00516	0.02166	0.01866
LC	12	0.00614	0.00176	0.0127	0.00446	0.01377	0.00528	0.01942	0.01719
SI	4	0.00651	0.00354	0.00865	0.0035	0.01095	0.0059	0.00788	0.00823

The low pairwise Fst values (range: −0.0070 to 0.0060) further support the absence of population structure among the sampling sites (Table 4). The negative values of pairwise Fst reported are treated as zero and are indicative of no differentiation.

**TABLE 4:** Pairwise Weir and Cockerham (1984) Fst between sampling locations for 88 individuals based on 20, 731 unlinked SNPs. Numbers in parentheses are the sample sizes for each location, while values in bold indicate Fst values significantly different from zero (95% confidence interval, 100 bootstraps).

	YCW (17)	ST (12)	SW (6)	LC (12)	BI (15)	SI (4)	PI (6)
ST (12)	0.0002						
SW (6)	0.0061	0.0008					
LC (12)	−0.0003	0.0010	0.0025				
BI (15)	0.0031	0.0022	0.0024	−0.0004			
SI (4)	0.0020	−0.0042	**−0.0039**	0.0001	−0.0024		
PI (6)	0.0045	0.0019	0.0057	0.0039	−0.0008	0.0022	
LM (18)	0.0034	0.0030	0.0018	0.0018	0.0000	−0.0070	−0.0030

### 3.3 Outlier Analyses

BayeScan and Outflank did not detect any outlier loci, while LFMM detected one outlier (426,354: 28). Since there were no outlier loci in common among these three methods, we did not report this as a candidate locus to minimise the chance of false positives. We detected 137 outlier SNPs based on the genome‐scan RDA method described in Capblancq et al. (2018) where SNPs were identified on their *p*‐value in the Mahalanobis distance distribution compared to the rest of the SNPs on the first two RDA axes, after correction for the genomic inflation factor (Luu et al. 2016) and adjusting for the false discovery rate (FDR) with *q*‐value threshold of 0.05 (Figures S5 and S6). Among the outlier SNPs, which show elevated differentiation relative to the background (Figure S7), 43 SNPs were mostly associated with pH, followed by 39 SNPs with Suspended Solids (SS), 25 SNPs with Temperature (TEMP), 20 SNPs with Dissolved Oxygen (DO) and 10 SNPs with Chlorophyll (CHL) levels (Table S4), although collinearity between these variables means that the correlations may be strong across multiple variables (Figure S5). The permutation test shows that the overall RDA model was significantly different from the null distribution (*F* = 1.0351, *p* = 0.001) and explained 5% of total variance. The first constrained RDA axis (RDA1) was significant (*F* = 1.1768, *p* = 0.001) but the further RDA axes (RDA2‐5) were insignificant. Hence, the environmental variables on RDA1 (positive association with CHL, pH, DO, TEMP, negative association with SS) explain a significant proportion of the constrained variance in the genotypes.

Of the 137 outlier loci identified, 95 were mapped to contigs in the *Oulastrea crispata* transcriptome. Of these, blast hits were returned for 93 (97.9%) while annotations with GO and InterPro GO terms were returned for 76 contigs (80%). Only transcripts that were the best hits for the RADseq loci and had overlapping GO and InterPro terms were further analysed, there were 54 unique GO terms identified, with 21 unique biological process, 20 molecular function and 13 cellular component GO terms (Tables S5 and S6). Functional annotation of the RDA outliers suggested that candidate loci are mainly associated with (i) external signal perception and transduction (e.g., transcription regulation, protein phosphorylation, signal transduction), (ii) membrane transport and ion homeostasis, particularly Ca^2+^−related transport, and (iii) ER/Golgi‐associated protein trafficking and cellular metabolism.

### 3.4 Symbiodiniaceae Endosymbionts Within *Oulastrea*

Across the 11 Symbiodiniaceae species investigated, *Durusdiunium trenchii* was the most highly represented across the water quality gradient (Low, Med, High) (Figure S8). The relative proportions of the read counts mapped to Symbiodiniaceae species differed significantly among the three water quality categories (PERMANOVA: *F* = 3.324, *p*‐value = 0.013). While the homogeneity of dispersion among water quality groups (betadisper) was not significant (*F* = 2.207, *p*‐value = 0.114), hence the assumption of equal group variance is satisfied for PERMANOVA. However, given the large discrepancy in number of reads between Durusdinium and the other Symbiodiniaceae tested, this variation in species composition within the host against water quality could reflect small shifts in the read numbers of the rarer species of the Symbiodiniaceae that may not be functionally relevant.

## 4 Discussion

Despite their proliferation across a well‐documented environmental gradient, we did not detect any spatial structuring of genetic variance in *Oulastrea crispata* (Figure 2). However, we detected 137 environment‐associated putative outlier loci based on the RDA analyses. Given that western waters are characterised by higher dissolved inorganic nitrogen, chlorophyll *a*, phosphate, and lower salinity that should exert selection pressure on coral survival, any adaptive response, if present, is limited to a small subset of loci rather than reflected in broad genome‐wide divergence. The lower observed heterozygosity compared to expected heterozygosity across sites (Table 3) is consistent with genetic drift or reduction in population size during the demographic history of *O. crispata* , rather than inbreeding (low Fis values; Table 3) or population structure (Figure 2), although these results should be interpreted with caution as RADseq can be biased towards the underestimation of heterozygosity from restriction‐site polymorphism and locus dropout, and can be confounded by other evolutionary changes. The pattern of very weak neutral population structure within our dataset as indicated by the low pairwise Fst values between sites (Table 4) resembles those found in other stress‐tolerant taxa, such as *Pocillopora damicornis* in the Great Barrier Reef (Torda et al. 2013) and *Porites lutea* in the South China Sea (Huang et al. 2018), where gene flow is mediated by long larval durations and generalist traits. In *Acropora* coral species, which are more environmentally sensitive, adaptive variation has been identified through strong allele frequency shifts and FST outlier analyses (Bay and Palumbi 2014; Thomas et al. 2022) as well as specific selective sweeps at particular loci (Leiva et al. 2025). In contrast, we did not detect the presence of FST outliers. Even in cases where population structure is absent, such as in *A. millepora* along the Great Barrier Reef, Fuller et al. (2020) found balancing selection on the heat shock protein sacsin, while bleaching susceptibility appears to be polygenic.

Pervasive gene flow within the small spatial scale of our dataset may have resulted in the homogenisation of the population. Low pairwise Fst values (Table 4) support high connectivity, indicating that dispersal and gene flow are sufficient to ensure mixing of alleles within the population. *O. crispata* is capable of both broadcast spawning (Zayasu et al. 2015) and brooding of asexual planula as a reproductive strategy (Nakano and Yamazato 1992; Lam 2000). Given the low Fis values across sites (Table 3: 0.0123–0.0232), outcrossing is likely to be the predominant reproductive mode. During the southwest monsoon season, prevailing coastal currents in Hong Kong can transport water masses over 50 km in under a week (Morton and Morton 1983), allowing larvae to reach and settle in distant, environmentally distinct locations. This passive dispersal may underlie the widespread genetic mixing observed across all sampled sites. The pattern of homogeneous population structure in the scale of 10‐1000 m has been mirrored in another spawning coral *Paracyathus stearnsii* as compared to another brooding coral, *Balanophyllia elegans* within the same range (Hellberg 1996), which supports sexual reproduction and broadcast spawning of *O. crispata* in Hong Kong despite their potential for brooding. The homogeneous population structure and levels of gene flow across locations for *O. crispata* in Hong Kong suggests that the metapopulation may be highly interconnected, although more sampling across the Indo‐Pacific range of *O. crispata* will be needed to confirm this.

There was mixed evidence of local adaptation in *O. crispata* to the water quality gradient in Hong Kong based on our dataset. Across the outlier detection and GEA methods used, the LFMM and *F*_ST_ outlier loci analyses did not identify overlapping signatures of selection, while the RDA model, which captured 5% of total variance, identified a subset of potential outliers (Table S4) that have annotated functions in detecting external stimuli and gene expression changes, pointing towards their putative roles as mechanisms for the broad environmental tolerance of *O. crispata* . The discrepancy among methods may reflect polygenic (multilocus) effects, as multivariate approaches like RDA may be better able to capture diffuse, multilocus signals relative to univariate methods like LFMM or standard outlier detection procedures (Forester et al. 2018). This is plausible, as traits related to environmental tolerance are likely quantitative and therefore may have a polygenic architecture, similar to the polygenic basis of bleaching susceptibility observed in *A. millepora* from the Great Barrier Reef (Fuller et al. 2020). However, these results should be interpreted with caution. Firstly, since environmental values were assigned at the site level, the effective level of replication in our RDA is the number of sites rather than the number of individuals. This substantially reduces the effective degrees of freedom and raises the risk of pseudoreplication, which can inflate Type I error (Hurlbert 1984). Secondly, as shown by Yeaman and Whitlock (2011), polygenic local adaptation is theoretically unlikely in a system with high connectivity, as each small effect locus is unlikely to be strong enough to prevent allele swamping. Thirdly, our sample size and site replication are limited, so the study may lack the power needed to resolve the subtle, coordinated allele‐frequency shifts expected under polygenic adaptation. These results should be interpreted as hypotheses to facilitate further study rather than definitive evidence of local adaptation in the current dataset in the absence of field/lab‐based sources of data to confirm this (Lowry et al. 2017).

The functional annotation of the outlier loci suggests a link between the candidate outlier loci and their roles in pathways relating to the environmental responses in *O. crsipata*. The annotated functions of the outlier loci (Table S5) are likely relevant to coral environmental responses, because signal transduction, ion transport, membrane trafficking, and metabolism are known to contribute to cellular homeostasis and calcification‐associated processes. Protein phosphorylation and carbohydrate metabolism have been implicated in the regulation of cnidaria‐algal symbiosis in *Aiptasia* (Hillyer et al. 2016; Simona et al. 2019) and may reflect shifts in energy budgeting under reduced light or eutrophic/turbid conditions. Ca^2+^ homeostasis and cation transport terms are consistent with processes underlying calcification in corals (Bernardet et al. 2019). In addition, the disruption of Ca^2+^ homeostasis is associated with stress response in corals (DeSalvo et al. 2008, 2010; Poquita‐Du et al. 2020). We therefore interpret these loci as candidate genomic regions associated with environmental response‐related cellular pathways, while recognising that transcriptome‐based annotation of short RAD loci is inferential and does not by itself demonstrate gene function or phenotypic plasticity.

If *O. crispata* is adapted to the environmental gradient studied in Hong Kong, a key question for future genetic studies is the genetic architecture underlying these adaptations. This question is informed by a growing body of work reporting signals of adaptation despite high connectivity in marine systems, including herring (Goodall et al. 2026), urchins (Rumberger et al. 2025), and molluscs (Lee et al. 2025). In theory, standing genetic variation can enable rapid responses to selection (Roff 1997), and many traits in nature have a polygenic basis (Bomblies and Peichel 2022). However, gene flow is expected to inhibit local adaptation, and in highly connected populations across environmental gradients, selection acting on small‐effect loci is likely too weak to produce detectable allele‐frequency changes (Cornwell 2020). Adaptation can still occur if it is supported by large‐effect loci or by genetic architecture that reduces recombination and increases the apparent effect of tightly linked variants (Yeaman and Whitlock 2011; Tigano and Friesen 2016), as illustrated by genetic architecture facilitating adaptation in herring (Goodall et al. 2026). With our RADseq dataset, we did not detect large‐effect loci or clear evidence of this architecture at the spatial scale examined. Nevertheless, adaptive variation may instead be driven by variants segregating across many genetic backgrounds, enabling rapid adaptation through “soft” selective sweeps (Messer and Petrov 2013), which can be difficult to resolve with reduced‐representation sequencing. Therefore, while our RDA outliers could represent such as signal, they remain putative and require validation. Future work could use a sampling design tailored to weak selection, such as paired‐gradient sampling (Dudaniec et al. 2018), and apply whole‐genome resequencing to better capture underlying genetic architecture.

The tolerance of *Oulastrea crispata* to broad environmental conditions may also be in part due to their stable association with *Durusdinium* spp., a genus of Symbiodiniaceae that is also tolerant to stressful conditions (LaJeunesse et al. 2014). *Durusdinium* spp. is the dominant symbiont in *O. crispata* across water quality gradients in Hong Kong, as indicated by reads mapping to *D. trenchii* occupying the highest proportion relative to other Symbiodiniaceae genomes (Figure S8). However, because *D. trenchii* is our only *Durusdinium* representative, these mapping results do not provide species‐level resolution within *Durusdinium* spp. Previous spatial and temporal scale observations by Chen et al. (2003) and Lien et al. (2007, 2013) indicate that the predominant *Durusdinium* strain associated with *O. crispata* tends to be *D. eurythalpos* in the tropics, while *D. boreaum* occurs in temperate regions, with a region of sympatry within the subtropics (LaJeunesse et al. 2014). In eastern oligotrophic waters of Hong Kong, *O. crispata* colonies harbour *D. eurythalpos* (Saad et al. 2021). Based on these prior reports, the strain in our samples is likely *D. eurythalpos*, though the consistency of this association within western sites has not been confirmed. Apart from *Durusdinium* as the main symbiont, the subtle but significant difference in species composition of other Symbiodiniaceae based on our RADseq reads suggests a source of variation could be investigated in further experiments as to whether some species are associated with environmental stress in *O. crispata* . The bacterial portion of the microbiome can also play a critical role in coral health and nutrient processing (Radecker et al. 2015; Voolstra et al. 2024), and a particular genus, *Ruegaria*, is a denitrifying bacteria and has been shown to help maintain healthy coral‐algal symbioses in a high nutrient environment (Xiang et al. 2026). Yet, 16S metabarcoding of the *O. crispata* microbiome showed relative stability of bacterial communities in individuals sampled across Hong Kong, with only 23% of the variation in coral microbiomes being explained by environmental differences (Röthig et al. 2020). This stands in contrast to corals like *Acropora* and *Tubastraea*, whose microbial assemblages shift dramatically in response to environmental stressors (Tandon et al. 2022; Girija et al. 2023) and may buffer against environmental selection of coral genotypes (Saad et al. 2021).

A relatively limited set of physiological studies have demonstrated that *O. crispata* individuals can exhibit high tolerance to broad environmental conditions, potentially via phenotypic plasticity as a hypothetical mechanism. At their poleward range edge, *O. crispata* exhibits tolerance to low temperatures between 7°C and 10°C (Yajima 1986) and a capacity to acclimate to short‐term temperature fluctuations in the laboratory (Keshavmurthy et al. 2021). Trophic studies of *O. crispata* in Hong Kong reveal that they are highly heterotrophic (Chei et al. 2025) and in the lab individuals have been shown to survive long periods in the dark (Denis et al. 2012), suggesting a pathway to survival in turbid, low light environments. Hence, the physiological flexibility of *O. crispata* allows for population persistence and can increase adaptation directly where phenotypic novelty becomes selected for (Levis et al. 2018), or indirectly by increasing time for natural selection to act on adaptive traits (Fox et al. 2019) but also restricts their capacity to adapt with climate change by reducing heritable variation (Snell‐Rood et al. 2018; Oostra et al. 2018), which can be seen in the lack of detectable genetic divergence in our dataset. A similar strategy of relying on phenotypic plasticity has also been recorded in another coral, *Porites astreoides* , that is highly resilient to environmental variable (Kenkel and Matz 2016).

While *O. crispata* exhibits high connectivity and weak neutral structure across its range, our study nevertheless detected candidate outlier loci via RDA that correlate with environmental variation. These loci represent preliminary leads for future investigation. While we do not have information to ascertain if they are adaptive loci, functional annotation suggests their possible roles in response to the environment. Besides the host genomic response, there could be other mechanisms that contribute to the resilience of *O. crispata* . These include a stable association with a stress‐tolerant endosymbiont, the generalist, flexible trophic strategies of this species, and its microbial community that allow individuals to survive across a spectrum of stress regimes. Such a strategy may offer selective advantages in rapidly changing or marginal reef environments, where local adaptation is less feasible or too slow to match the pace of disturbance. These life history traits and physiological flexibility underscore its potential as a model organism for exploring coral resilience under marginal conditions (Röthig et al. 2020). While not a focal species itself, these findings can be informative for restoration efforts that integrate the conservation of genetic diversity. Future work to detect barriers to gene flow and investigate the broad environmental tolerance across the species range *O. crispata* could adopt a whole genome resolution to detecting and validating adaptive loci (Selmoni et al. 2024) and include their epigenetic potential (Sheldon et al. 2025). Coupling these data with biophysical models of larval transport (Bode et al. 2019) could also illuminate how oceanographic processes shape population connectivity. This positions *O. crispata* as a useful system for studying how genetic and non‐genetic mechanisms contribute to coral resilience in the face of climate change and coastal disturbance.

## Author Contributions

**Le Qin Choo:** data curation (lead), formal analysis (equal), writing – original draft (equal), writing – review and editing (equal). **Vriko Yu:** conceptualization (equal), investigation (equal), writing – original draft (equal), writing – review and editing (equal). **Paolo Momigliano:** formal analysis (equal), writing – review and editing (equal). **Shelby E. McIlroy:** conceptualization (equal), investigation (equal), writing – original draft (equal), writing – review and editing (equal).

## Funding

Funding for this research was provided by the Research Grants Council of Hong Kong (GRF‐17113923) and The Ocean Park Conservation Foundation Hong Kong (Funding Reference: OT01.1718).

## Conflicts of Interest

The authors declare no conflicts of interest.

## Supporting information


**Table S1:** List of assembled genomes of the class Dinophyceae included for taxonomic classification of catalogue loci.
**Table S2:** K‐values and cross‐validation (CV) error (1–5) for the admixture analysis.
**Table S3:** Number of variant and fixed loci per sampling location used in population diversity calculations.
**Table S4:** RDA outliers, associated loadings and correlated environmental variables.
**Table S5:** List and frequency of GO terms identified from outlier loci.
**Table S6:** Outlier RADseq locus and SNP from RDA analysis, associated environmental variable, best‐hit transcript identified and description of transcript from Blast2GO.
**Figure S1:** Pipeline for *Oulastrea crispata* RADseq processing and population genomics filtering and analyses.
**Figure S2:** Principal component analysis (PCA) plots based on 39,527 SNPs from 90 individuals of *Oulastrea crispata* collected across Hong Kong. Each point is coloured according to their individual missingness across the SNP dataset.
**Figure S3:** Pairwise co‐ancestry plot based on 81 individuals of *O. crispata* , excluding individuals with more than 20% missing sites. Darker colours indicate high levels of relatedness between individuals.
**Figure S4:** Admixture chromplot of *K* = 2–5 for 90 *Oulastrea crispata* individuals grouped according to their sampling location. Colours of each bar indicate assignment into an ancestral population.
**Figure S5:** Redundancy analysis (RDA) bi‐plot for the MAF filtered dataset of 20,731 SNPs, where grey circles indicate SNPs, coloured circles indicate individuals coloured according to their grouping (Top: water quality categories, Below: sampling location) and the arrows indicate the environmental parameters included after VIF filtering: CHL, chlorphophyll *a*, DO, dissolved oxygen; SS, suspended solids; TEMP, temperature, and pH.
**Figure S6:** Identification of 137 outlier SNPs using the RDA genome‐scan approach from Capblancq et al. (2018). The SNPs highlighted in orange are the outliers (q‐threshold = 0.05) that are shared between the first two RDA axes that represent 43% of constrained variance attributed to environmental predictors in the dataset. Top: The plot above shows the outlier loci in a Manhattan plot, where the loci have been arranged in numerical order. Below: The outlier SNPs are highlighted in orange in the RDA bi‐plot.
**Figure S7:** FST distributions for the identified outlier loci (*n* = 137) relative to the remaining loci (*n* = 20,594) for (A) Water quality and (B) Sampling locations. Higher FST differentiation is observed in the identified outlier SNPs as compared to the remaining SNPs when grouped by water quality (Welch's *t*‐test: *t* = 16.455, df = 136.58, *p*‐value < 2.2e‐16) or sampling locations (Welch's t‐test: *t* = 20.22, df = 137.52, *p*‐value < 2.2e‐16).
**Figure S8:** Boxplots of the percentage (above) and log(10) transformed percentage (below) of unambiguously mapped RADseq readsmapped to each Symbiodinaeceae reference genome, across the water quality gradient (Low, Medium and High). Note the log‐scaled y‐axis (below) for better visualisation as reads mapped are low across all Symbiodinaceae except for *Durusdinium trenchii*.

**Data S1:** File of unambiguous reads mapping to each Symbiodiniaceae reference across each individual.

## Data Availability

RAD sequences are deposited on NCBI as BioProject PRJNA1389676. The scripts used to generate results from the analyses are accessible on Zenodo with the following DOI 10.5281/zenodo.17983077.
