# Supplementary material for: Weak Structure and Environment‐Associated Loci Across a Eutrophication Gradient in a Resilient Coral Species
Source: Ecol Evol. 2026 Jul 1;16(7):e73908. doi: 10.1002/ece3.73908 (PMC13322662; doi:10.1002/ece3.73908)

**Supplementary Materials**

Table S1: List of assembled genomes of the class Dinophyceae included for taxonomic classification of catalogue loci

| Assembly Accession | Assembly Name | Organism Name | Organism Infraspecific Names Strain | Assembly Level | Assembly Release Date | WGS project accession | Assembly Stats Number of Scaffolds | Mapped to transcriptome (Quek & Huang, 2019) |
| --- | --- | --- | --- | --- | --- | --- | --- | --- |
| GCA_964212085.1 | pySymPilo4.1 | *Symbiodinium pilosum* |  | Chromosome | 3/8/2024 | CAXOTO01 | 1816 | Y |
| GCA_000507305.1 | ASM50730v1 | *Breviolum minutum Mf 1.05b.01* | Mf 1.05b.01 | Scaffold | 17/7/2013 | BASF01 | 21899 | Y |
| GCA_001939145.1 | ASM193914v1 | *Symbiodinium microadriaticum* | CCMP2467 | Scaffold | 6/1/2017 | LSRX01 | 9688 | Y |
| GCA_003297005.1 | SymA ver 1.0 | *Symbiodinium* sp*. clade A Y106* | Y106 | Scaffold | 22/6/2018 | BGNK01 | 16176 | Y |
| GCA_003297045.1 | SymC ver 1.0 | *Symbiodinium* sp*. clade C Y103* | Y103 | Scaffold | 22/6/2018 | BGPT01 | 6576 | Y |
| GCA_009767595.1 | ASM976759v1 | *Symbiodinium kawagutii* | SL-2019 | Scaffold | 18/12/2019 | VSDK01 | 30040 | N |
| GCA_037576205.1 | ASM3757620v1 | *Pyrocystis lunula* | IRGN E6MV7HEDUA | Scaffold | 26/3/2024 | JAPIWU01 | 447525 | N |
| GCA_037575995.1 | ASM3757599v1 | *Lingulodinium polyedra* | IRGN 42FCANL8J5 | Scaffold | 26/3/2024 | JAOYNT01 | 224392 | N |
| GCA_947184155.2 | Cgoreaui_SCF055-01_v2.1 | *Cladocopium goreaui* |  | Scaffold | 6/4/2024 | CAMXCT02 | 6843 | Y |
| GCA_963575745.1 | Prorocentrum_cordatum_CCMP1329 | *Polarella glacialis* |  | Scaffold | 5/11/2023 | CAUYUJ01 | 22724 | N |
| GCA_019702695.1 | ASM1970269v1 | *Amphidinium carterae* | NMCA1314 | Scaffold | 20/8/2021 | JAAMXW01 | 977563 | N |
| GCA_963377175.1 | EvRCC1521 | *Effrenium voratum* |  | Scaffold | 24/8/2023 | CAUJNA01 | 3881 | N |
| GCA_905221605.1 | Snat_CCMP2548 | *Symbiodinium natans* |  | Scaffold | 20/2/2021 | CAJNDS01 | 2855 | Y |
| GCA_905221615.1 | Stri_CCMP2592 | *Symbiodinium* sp*. CCMP2592* |  | Scaffold | 20/2/2021 | CAJNDT01 | 6245 | Y |
| GCA_905178155.1 | a120_v1 | *Amoebophrya* sp*. A120* |  | Scaffold | 28/9/2021 | CAJINV01 | 401 | N |
| GCA_905178165.1 | a25_v1 | *Amoebophrya* sp*. A25* |  | Scaffold | 28/9/2021 | CAJINU01 | 597 | N |
| GCA_963970005.1 | Durusdinium_trenchii_CCMP2556 | *Durusdinium trenchii* |  | Scaffold | 4/3/2024 | CAXAMN01 | 29137 | Y |
| GCA_005223375.1 | ASM522337v1 | *Amoebophrya* sp*. AT5.2* | AT5.2 | Contig | 7/5/2019 | RXOD01 | 13796 | N |
| GCA_905231915.1 | Snec_CCMP2469 | *Symbiodinium necroappetens* |  | Scaffold | 20/2/2021 | CAJNJA01 | 104583 | Y |
| GCA_905221635.1 | Slin_CCMP2456 | *Symbiodinium* sp*. CCMP2456* |  | Scaffold | 20/2/2021 | CAJNDV01 | 37772 | Y |

Table S2: K-values and cross-validation (CV) error (1-5) for the admixture analysis.

| K | CV error |
| --- | --- |
| 1 | 0.55259 |
| 2 | 0.59626 |
| 3 | 0.63875 |
| 4 | 0.70835 |
| 5 | 0.76327 |

Table S3: Number of variant and fixed loci per sampling location used in population diversity calculations

| # Pop ID | Private | Sites | Variant Sites | Polymorphic Sites | %Polymorphic Loci | No. individuals |
| --- | --- | --- | --- | --- | --- | --- |
| YCW | 134599 | 29890469 | 2093325 | 1272167 | 4.2561 | 17 |
| ST | 103516 | 30840953 | 2101926 | 1110481 | 3.60067 | 12 |
| PI | 64914 | 29968516 | 2060182 | 849353 | 2.83415 | 6 |
| LM | 133646 | 26504187 | 1985206 | 1233805 | 4.65513 | 18 |
| SW | 46724 | 25878109 | 1885835 | 700745 | 2.70787 | 6 |
| BI | 147467 | 31643829 | 2149522 | 1296145 | 4.09604 | 15 |
| LC | 140258 | 34191082 | 2217226 | 1229783 | 3.59679 | 12 |
| SI | 55064 | 35447717 | 2202465 | 607306 | 1.71324 | 4 |

Table S4: RDA outliers, associated loadings and correlated environmental variables

| Locus: SNP | RDA1 | RDA2 | RDA3 | RDA4 | RDA5 | Predictor | Correlation |
| --- | --- | --- | --- | --- | --- | --- | --- |
| 100386:36 | -0.02687 | -0.00128 | -0.00241 | -0.00304 | 0.015798 | SS | 0.313734979 |
| 10835:38 | -0.02532 | -0.00774 | -0.00667 | -0.00518 | 0.002009 | SS | 0.268754847 |
| 108960:9 | -0.02115 | -0.0147 | -0.00387 | -0.01183 | -0.00416 | SS | 0.162276967 |
| 110336:88 | -0.01579 | -0.02219 | 0.014736 | 0.012241 | -0.00268 | TEMP | 0.130841317 |
| 113179:11 | -0.00672 | 0.02782 | -0.00252 | -0.01091 | 0.006683 | SS | 0.213656123 |
| 114464:22 | -0.0203 | 0.015726 | 0.004281 | -0.00054 | -0.00394 | pH | 0.337093821 |
| 114855:108 | -0.01636 | 0.032426 | -0.00804 | -0.00619 | -0.00813 | pH | 0.329941134 |
| 1162:35 | -0.01673 | 0.021258 | 0.002011 | -0.00169 | -0.00607 | pH | 0.298229382 |
| 11780:110 | -0.00346 | -0.0299 | 0.004954 | -0.00127 | 0.007642 | TEMP | 0.205255596 |
| 118377:86 | -0.02978 | 0.005592 | 0.005128 | 0.018083 | 0.005604 | DO | 0.404577962 |
| 120325:113 | -0.02287 | 0.023887 | 0.007925 | 0.000821 | -0.02049 | pH | 0.382871608 |
| 123239:83 | 0.028395 | -0.01932 | 0.007822 | -0.00866 | -0.00317 | pH | 0.367020752 |
| 125926:27 | -0.01545 | -0.0264 | -0.00372 | -0.0055 | -0.0016 | CHL | 0.087823486 |
| 126424:102 | -0.02727 | -0.01084 | -0.00214 | 0.002711 | -0.00488 | pH | 0.228797946 |
| 131785:113 | 0.021055 | 0.013481 | 0.003887 | 0.000829 | 0.001168 | SS | 0.178723831 |
| 133647:40 | -0.02224 | 0.018025 | 0.006225 | 0.012682 | -0.00334 | DO | 0.388530223 |
| 133833:93 | -0.03043 | 0.000804 | 0.002206 | -0.01196 | 0.005076 | SS | 0.37151455 |
| 137268:98 | -0.02533 | -0.01037 | 0.001704 | -0.00881 | 0.005033 | SS | 0.303721906 |
| 141413:114 | -0.02184 | 0.013677 | 0.004058 | -0.00618 | -0.00611 | pH | 0.307966538 |
| 142718:33 | -0.02028 | 0.018038 | 0.008066 | 0.007802 | -0.00549 | DO | 0.349826791 |
| 144290:110 | -0.03015 | 0.002864 | 0.015482 | -0.01413 | 0.002372 | SS | 0.3470306 |
| 14540:63 | -0.00258 | 0.028991 | -0.0031 | 0.005037 | 0.002162 | TEMP | 0.245596486 |
| 146758:50 | -0.02985 | 0.019421 | -0.00136 | -0.00212 | -0.00727 | pH | 0.386841036 |
| 147049:57 | -0.00599 | -0.02602 | 0.00268 | 0.006693 | 0.020536 | TEMP | 0.147276913 |
| 148494:102 | -0.02489 | -0.00637 | 0.009613 | -0.00847 | -0.01636 | pH | 0.29002574 |
| 150259:77 | -0.00526 | 0.024224 | 0.000247 | 0.013171 | 0.014858 | DO | 0.250535168 |
| 15048:46 | -0.02727 | -0.00185 | -0.0046 | 0.007853 | 0.001664 | SS | 0.274028184 |
| 150725:109 | -0.02636 | -0.02891 | -0.01202 | 0.007002 | 0.005717 | CHL | 0.181626468 |
| 15148:79 | -0.02937 | -0.00047 | 0.00415 | 0.00051 | -0.00207 | pH | 0.365130074 |
| 151998:115 | -0.02723 | 0.002186 | 0.01057 | 0.000344 | -0.01121 | pH | 0.421150238 |
| 15565:91 | -0.00701 | -0.0267 | 0.010355 | -0.01429 | -0.00796 | TEMP | 0.255453199 |
| 15581:80 | -0.0255 | 0.005802 | -0.00142 | 0.00713 | -0.01158 | pH | 0.291346967 |
| 15713:90 | -0.00084 | 0.027665 | -8.49E-05 | 0.001111 | -0.0087 | TEMP | 0.160383388 |
| 15737:67 | 0.005671 | 0.023848 | 0.012138 | -0.01692 | 0.016576 | CHL | 0.309110243 |
| 157993:69 | -0.02434 | -0.01174 | 0.011075 | -0.01523 | 0.012958 | SS | 0.294882877 |
| 158956:97 | 0.023978 | -0.00584 | 0.004046 | -0.00755 | -0.00248 | SS | 0.300536989 |
| 159217:82 | 0.010423 | 0.023251 | 0.000732 | 0.007638 | 0.002696 | TEMP | 0.10236879 |
| 159566:72 | 0.011947 | -0.02633 | 0.014227 | -0.00056 | -0.01118 | TEMP | 0.277488209 |
| 161193:114 | -0.02433 | 0.012154 | -0.00582 | -0.02105 | -0.00588 | pH | 0.292117358 |
| 161377:85 | -0.01032 | 0.027779 | 0.002452 | 0.00666 | 0.000785 | DO | 0.295290523 |
| 161710:56 | -0.02698 | 0.002561 | -0.00843 | 0.005731 | 0.005896 | SS | 0.287360296 |
| 162654:105 | 0.01732 | -0.02249 | 0.009706 | -0.00611 | 0.002869 | TEMP | 0.346334779 |
| 162886:51 | -0.03036 | 0.017077 | 0.014619 | 0.012977 | -0.01821 | DO | 0.451785824 |
| 16326:8 | -0.03181 | -0.00539 | 0.000354 | 0.007625 | 0.01064 | SS | 0.325489985 |
| 168841:13 | -0.01987 | -0.01711 | -0.0108 | -0.0084 | 0.003708 | SS | 0.195216487 |
| 169364:79 | -0.0191 | 0.01713 | 0.015353 | 0.004991 | 0.005191 | DO | 0.333686705 |
| 170995:79 | 0.017853 | -0.02161 | -0.01297 | 0.002841 | 0.005407 | pH | 0.37327093 |
| 172491:51 | -0.03048 | -0.01581 | 0.007454 | 0.002259 | 0.003453 | SS | 0.293461748 |
| 172952:91 | 0.013162 | -0.02615 | -0.00078 | 0.010838 | 0.016717 | pH | 0.288171311 |
| 173297:120 | 0.010648 | 0.021534 | 0.000821 | 0.010521 | -0.01075 | TEMP | 0.12832102 |
| 17332:25 | 0.031544 | 0.002565 | -0.00406 | -0.01779 | -0.00148 | DO | 0.312505867 |
| 17547:17 | -0.02957 | -0.00268 | 0.012716 | 0.012967 | -0.00486 | DO | 0.342825921 |
| 177720:27 | 0.011346 | 0.021911 | -0.00723 | 0.001329 | 0.000295 | TEMP | 0.174118874 |
| 180634:95 | -0.0328 | 0.019913 | -0.0169 | 6.10E-05 | 0.008114 | SS | 0.393585707 |
| 18183:105 | -0.02747 | -0.00251 | -0.0098 | -0.01471 | -0.0055 | SS | 0.263568938 |
| 183133:27 | 0.020876 | 0.012552 | -0.00717 | 0.00094 | -0.00039 | SS | 0.216685304 |
| 184071:24 | 0.012341 | 0.021451 | 0.003708 | -0.00964 | -0.0037 | CHL | 0.142550602 |
| 184357:114 | -0.01701 | -0.02164 | -0.00275 | 0.007334 | 0.00737 | SS | 0.127001628 |
| 18647:43 | -0.02855 | -0.00277 | 0.009462 | -0.0141 | 0.018307 | SS | 0.409281855 |
| 187214:78 | -0.00265 | -0.02677 | -0.00177 | 0.002607 | -0.00294 | DO | 0.119271973 |
| 18768:118 | -0.0043 | 0.027586 | -0.0056 | -0.00748 | -0.01282 | TEMP | 0.214151634 |
| 19143:59 | -0.00418 | -0.02654 | -0.01591 | 0.00783 | 0.003355 | DO | 0.165676098 |
| 19163:25 | -0.02522 | 0.006775 | -0.00048 | 0.014999 | -0.02196 | CHL | 0.418461948 |
| 20310:80 | -0.02809 | -0.01053 | -0.01797 | 0.008598 | -0.00339 | CHL | 0.284478233 |
| 204376:29 | -0.02667 | -0.00867 | -0.00763 | -0.01047 | -0.0037 | SS | 0.256044746 |
| 205143:33 | -0.00096 | -0.02783 | -0.01891 | -0.002 | -0.00155 | DO | 0.225336949 |
| 214315:108 | 0.02798 | -0.008 | 0.008149 | 0.006777 | 0.000576 | SS | 0.342051827 |
| 214345:9 | -0.01127 | -0.02361 | -0.00298 | 0.001262 | 0.00814 | TEMP | 0.068292716 |
| 220490:54 | -0.00888 | -0.02869 | -0.00985 | -0.01034 | -0.01002 | DO | 0.160260802 |
| 22168:21 | -0.02585 | -0.00788 | -0.00274 | -0.01743 | 0.010724 | SS | 0.307371316 |
| 22296:60 | -0.01925 | 0.017342 | -0.00179 | 0.008987 | -0.01144 | pH | 0.275171076 |
| 22785:17 | 0.020626 | -0.01469 | 0.003458 | 0.004301 | 0.01238 | pH | 0.341518534 |
| 22893:18 | -0.01786 | -0.01903 | 0.004273 | -0.01 | -0.00846 | pH | 0.142097318 |
| 2316:70 | -0.00908 | 0.02425 | -0.00706 | 0.008752 | 0.016325 | TEMP | 0.308798279 |
| 23173:49 | -0.02656 | 0.001235 | 0.001904 | 0.004904 | -0.0055 | pH | 0.348500615 |
| 234978:64 | -0.02268 | -0.01273 | 0.007874 | 0.003803 | -0.01133 | pH | 0.223208047 |
| 243314:49 | -0.02638 | -0.00588 | 0.008863 | -0.01374 | 0.01846 | SS | 0.333099834 |
| 24364:102 | -0.00439 | 0.02421 | 0.004294 | 0.004694 | 0.005495 | DO | 0.355491756 |
| 24538:117 | -0.02527 | -0.01154 | 0.013042 | 0.000188 | -0.00831 | pH | 0.245253823 |
| 245478:93 | 0.002094 | -0.02767 | 0.004036 | -0.00244 | 0.006228 | TEMP | 0.209403615 |
| 246561:92 | -0.02576 | 0.00558 | 0.000707 | -0.00132 | -0.00421 | pH | 0.320354114 |
| 2465689:79 | 0.014286 | 0.019191 | -0.00546 | -0.01637 | 0.009517 | CHL | 0.260371525 |
| 25128:9 | -0.0268 | -0.00555 | 0.010377 | 0.008367 | -0.01035 | pH | 0.265292261 |
| 251732:29 | -0.02214 | 0.013856 | -0.00821 | -0.00628 | 0.00704 | SS | 0.286895029 |
| 255409:45 | -0.02173 | 0.018174 | -0.00117 | 0.004631 | -0.00017 | pH | 0.346741687 |
| 25602:29 | 0.021508 | -0.01518 | 0.00289 | -0.00848 | -0.00118 | DO | 0.256800352 |
| 25750:84 | 0.013907 | -0.0216 | 0.010751 | 0.000843 | -0.00108 | TEMP | 0.285702405 |
| 258513:19 | -0.02544 | 0.009863 | -0.00254 | 0.000294 | -0.00221 | pH | 0.326047464 |
| 2641:17 | -0.02715 | -0.01033 | 0.000129 | 0.012224 | -0.01637 | CHL | 0.32075495 |
| 264674:75 | -0.02728 | 0.001153 | -0.00229 | -0.00735 | -0.01218 | pH | 0.291831869 |
| 2670:76 | -0.02545 | -0.00929 | 0.002756 | -0.00922 | 0.005599 | SS | 0.388795597 |
| 275330:109 | -0.02425 | -0.02292 | -0.00309 | -0.01077 | 0.002881 | SS | 0.171212826 |
| 276634:48 | -0.02333 | -0.02736 | -0.01658 | 0.001143 | 0.028026 | SS | 0.22424364 |
| 3054:120 | -0.02676 | -0.00457 | -0.0008 | -0.00303 | -0.01472 | pH | 0.28797798 |
| 30593:9 | -0.02419 | -0.01434 | 0.007901 | -0.0142 | 0.000438 | SS | 0.291330715 |
| 3102630:79 | 0.027444 | -0.00168 | -0.00636 | -0.01147 | -0.00136 | DO | 0.354800385 |
| 310773:71 | -0.0137 | 0.026749 | 0.000932 | -0.01588 | 0.002859 | pH | 0.270087066 |
| 321012:117 | 0.03008 | -0.01299 | 0.000583 | 0.003132 | -0.0166 | SS | 0.413153206 |
| 33534:72 | 0.004749 | 0.024822 | 0.007335 | -0.00653 | 0.013998 | CHL | 0.229066759 |
| 389907:11 | -0.02479 | -0.00681 | -0.02255 | 0.009783 | 0.010262 | TEMP | 0.286959244 |
| 399930:100 | 0.001919 | 0.024405 | -0.00996 | 0.003591 | -0.00026 | TEMP | 0.220998626 |
| 43356:41 | -0.02604 | 0.001805 | 0.000707 | -0.0076 | -0.01752 | pH | 0.291375964 |
| 43414:5 | 0.012234 | -0.02401 | 0.00176 | 0.000931 | 0.008006 | pH | 0.322993047 |
| 44183:38 | 0.001912 | 0.024157 | -0.00468 | 0.012419 | 0.002642 | TEMP | 0.191396227 |
| 50096:71 | 0.00535 | 0.023932 | 0.000262 | 0.011156 | 0.006101 | DO | 0.158379131 |
| 55363:84 | 0.02508 | 0.01437 | -0.01077 | -0.00303 | -0.0221 | SS | 0.261306034 |
| 56814:111 | -0.02667 | -0.01844 | -0.00521 | -0.00695 | -0.00436 | SS | 0.184790986 |
| 57278:70 | -0.01281 | -0.0282 | -0.00063 | 0.006784 | 0.021965 | SS | 0.101528975 |
| 57901:93 | 0.025867 | -0.00846 | 0.012091 | -0.00687 | -0.00298 | TEMP | 0.311755595 |
| 626359:106 | 0.013135 | -0.0238 | -0.00207 | 0.002326 | -0.00591 | SS | 0.276499968 |
| 65722:87 | -0.01617 | 0.019775 | 0.011431 | -0.0053 | 0.00119 | pH | 0.327093278 |
| 66050:62 | -0.01478 | -0.0241 | 0.003498 | -0.00505 | -0.00453 | TEMP | 0.107616359 |
| 66664:20 | 0.013444 | -0.02347 | 0.017915 | 0.004656 | 0.005852 | TEMP | 0.335719122 |
| 67815:93 | -0.02378 | -0.01178 | 0.001173 | -0.0067 | -0.0023 | SS | 0.206425218 |
| 73161:78 | -0.0267 | -0.0055 | -0.00438 | -0.00873 | 7.32E-05 | SS | 0.287172378 |
| 74234:58 | 0.022267 | 0.020252 | 0.007958 | -0.00559 | -0.00276 | CHL | 0.153488863 |
| 75212:91 | -0.03077 | -0.00045 | -0.00226 | -0.00491 | -0.02209 | pH | 0.371663737 |
| 75306:105 | 0.027403 | -0.00057 | 0.004971 | -0.00257 | -0.01 | SS | 0.302447231 |
| 75625:96 | -0.02567 | 0.011568 | -0.00984 | -0.00574 | -0.00523 | pH | 0.30037956 |
| 78255:15 | -0.02362 | 0.010579 | -0.01958 | 0.019569 | -0.007 | TEMP | 0.405619473 |
| 79806:30 | -0.02092 | 0.014905 | -0.00225 | -0.01184 | -0.0088 | pH | 0.299919797 |
| 80751:102 | -0.02314 | -0.01197 | 0.011348 | -0.01037 | -0.01062 | pH | 0.237230517 |
| 82675:7 | 0.02559 | 0.00187 | -0.00842 | -0.00631 | -0.00836 | SS | 0.314656149 |
| 83351:85 | -0.02824 | 0.010476 | 0.009258 | 0.00344 | -0.00509 | pH | 0.350782561 |
| 85300:17 | -0.00792 | -0.02517 | 0.000177 | -0.00676 | 0.016703 | TEMP | 0.148524761 |
| 87458:50 | -0.03307 | 0.000276 | 0.011808 | 0.022259 | 0.011562 | DO | 0.398599709 |
| 89050:15 | 0.002406 | 0.023594 | 0.008687 | -0.00509 | 0.003741 | DO | 0.1337784 |
| 89967:108 | -0.02576 | 0.016472 | -0.00382 | 0.001349 | 0.002535 | pH | 0.304387303 |
| 90388:71 | -0.00046 | 0.025506 | 0.013163 | 0.004429 | -0.00353 | DO | 0.240064742 |
| 90598:18 | -0.02018 | 0.019557 | 0.000592 | 0.003243 | -0.00266 | pH | 0.383755585 |
| 9068:68 | -0.00948 | 0.02315 | -0.00157 | -0.00872 | -0.00483 | pH | 0.265415494 |
| 91567:28 | 0.030716 | -0.01417 | 0.00819 | -0.00714 | -0.0014 | pH | 0.417200691 |
| 92121:20 | -0.00532 | -0.02662 | 0.020034 | -0.00847 | -0.01002 | TEMP | 0.276026576 |
| 92829:63 | -0.01122 | 0.029258 | 0.007529 | -0.00344 | 0.005827 | pH | 0.295996047 |
| 95120:79 | -0.02668 | -0.00516 | 0.005739 | -0.02374 | 0.000243 | SS | 0.287384246 |
| 96610:5 | -0.03068 | -0.00509 | -0.00042 | -0.00011 | -0.00721 | pH | 0.321653486 |
| 97176:60 | -0.03202 | 0.007331 | -0.0035 | -0.00364 | -0.00836 | pH | 0.417254768 |

Table S5: List and frequency of GO terms identified from outlier loci

| Frequency | Category | GO term | TERM |
| --- | --- | --- | --- |
| 6 | Cellular Component (C) | GO:0005886 | plasma membrane |
| 3 | Cellular Component (C) | GO:0016020 | membrane |
| 3 | Molecular Function (F) | GO:0005412 | glucose:sodium symporter activity |
| 3 | Molecular Function (F) | GO:0005524 | ATP binding |
| 2 | Cellular Component (C) | GO:0005789 | endoplasmic reticulum membrane |
| 2 | Molecular Function (F) | GO:0004930 | G protein-coupled receptor activity |
| 2 | Molecular Function (F) | GO:0005085 | guanyl-nucleotide exchange factor activity |
| 2 | Molecular Function (F) | GO:0005515 | protein binding |
| 2 | Molecular Function (F) | GO:0015179 | L-amino acid transmembrane transporter activity |
| 2 | Molecular Function (F) | GO:0031267 | small GTPase binding |
| 2 | Biological Process (P) | GO:0006468 | protein phosphorylation |
| 2 | Biological Process (P) | GO:0007186 | G protein-coupled receptor signaling pathway |
| 1 | Cellular Component (C) | GO:0005737 | cytoplasm |
| 1 | Cellular Component (C) | GO:0005743 | mitochondrial inner membrane |
| 1 | Cellular Component (C) | GO:0005783 | endoplasmic reticulum |
| 1 | Cellular Component (C) | GO:0005794 | Golgi apparatus |
| 1 | Cellular Component (C) | GO:0005819 | spindle |
| 1 | Cellular Component (C) | GO:0009986 | cell surface |
| 1 | Cellular Component (C) | GO:0031012 | extracellular matrix |
| 1 | Cellular Component (C) | GO:0032991 | protein-containing complex |
| 1 | Cellular Component (C) | GO:0044613 | nuclear pore central transport channel |
| 1 | Cellular Component (C) | GO:0070971 | endoplasmic reticulum exit site |
| 1 | Molecular Function (F) | GO:0000026 | alpha-1,2-mannosyltransferase activity |
| 1 | Molecular Function (F) | GO:0003676 | nucleic acid binding |
| 1 | Molecular Function (F) | GO:0004672 | protein kinase activity |
| 1 | Molecular Function (F) | GO:0005509 | calcium ion binding |
| 1 | Molecular Function (F) | GO:0008083 | growth factor activity |
| 1 | Molecular Function (F) | GO:0008198 | ferrous iron binding |
| 1 | Molecular Function (F) | GO:0008270 | zinc ion binding |
| 1 | Molecular Function (F) | GO:0015662 | P-type ion transporter activity |
| 1 | Molecular Function (F) | GO:0017056 | structural constituent of nuclear pore |
| 1 | Molecular Function (F) | GO:0019829 | ATPase-coupled monoatomic cation transmembrane transporter activity |
| 1 | Molecular Function (F) | GO:0030276 | clathrin binding |
| 1 | Molecular Function (F) | GO:0042802 | identical protein binding |
| 1 | Molecular Function (F) | GO:0061630 | ubiquitin protein ligase activity |
| 1 | Biological Process (P) | GO:0000132 | establishment of mitotic spindle orientation |
| 1 | Biological Process (P) | GO:0005975 | carbohydrate metabolic process |
| 1 | Biological Process (P) | GO:0006357 | regulation of transcription by RNA polymerase II |
| 1 | Biological Process (P) | GO:0006506 | GPI anchor biosynthetic process |
| 1 | Biological Process (P) | GO:0006607 | NLS-bearing protein import into nucleus |
| 1 | Biological Process (P) | GO:0006874 | intracellular calcium ion homeostasis |
| 1 | Biological Process (P) | GO:0006886 | intracellular protein transport |
| 1 | Biological Process (P) | GO:0006999 | nuclear pore organization |
| 1 | Biological Process (P) | GO:0007165 | signal transduction |
| 1 | Biological Process (P) | GO:0007166 | cell surface receptor signaling pathway |
| 1 | Biological Process (P) | GO:0016192 | vesicle-mediated transport |
| 1 | Biological Process (P) | GO:0016477 | cell migration |
| 1 | Biological Process (P) | GO:0031179 | peptide modification |
| 1 | Biological Process (P) | GO:0036228 | protein localization to nuclear inner membrane |
| 1 | Biological Process (P) | GO:0048208 | COPII vesicle coating |
| 1 | Biological Process (P) | GO:0055085 | transmembrane transport |
| 1 | Biological Process (P) | GO:0061172 | regulation of establishment of bipolar cell polarity |
| 1 | Biological Process (P) | GO:0090263 | positive regulation of canonical Wnt signaling pathway |
| 1 | Biological Process (P) | GO:1905475 | regulation of protein localization to membrane |

Table S6: Outlier RADseq locus and SNP from RDA analysis, associated environmental variable, best-hit transcript identified and description of transcript from Blast2GO.

| RAD Locus:SNP | Env variable | Best blast hit Transcript | Description |
| --- | --- | --- | --- |
| 18183:105 | SS | Oulastrea_crispata_104028 | sodium/mannose cotransporter SLC5A10-like |
| 78255:15 | TEMP | Oulastrea_crispata_38528 | adhesion G protein-coupled receptor L3-like |
| 57278:70 | SS | Oulastrea_crispata_59857 | putative E3 ubiquitin-protein ligase UBR7 |
| 137268:98 | SS | Oulastrea_crispata_121013 | PHD finger protein 14-like |
| 1162:35 | pH | Oulastrea_crispata_117484 | endoplasmic reticulum transmembrane helix translocase-like |
| 44183:38 | TEMP | Oulastrea_crispata_72578 | IQ motif and ankyrin repeat domain-containing protein 1-like |
| 65722:87 | pH | Oulastrea_crispata_38926 | uncharacterized protein LOC144635955 |
| 3054:120 | pH | Oulastrea_crispata_69769 | glypican-5-like |
| 15713:90 | TEMP | Oulastrea_crispata_26055 | GPI mannosyltransferase 4-like |
| 180634:95 | SS | Oulastrea_crispata_87263 | ankyrin-3-like isoform X2 |
| 125926:27 | CHL | Oulastrea_crispata_4623 | octopamine receptor 1-like |
| 184357:114 | SS | Oulastrea_crispata_76395 | TATA element modulatory factor-like |
| 214345:9 | TEMP | Oulastrea_crispata_81080 | AP-3 complex subunit beta-2-like |
| 276634:48 | SS | Oulastrea_crispata_75489 | protein TFG-like |
| 173297:120 | TEMP | Oulastrea_crispata_33776 | mitochondrial coenzyme A transporter SLC25A42-like |
| 18183:105 | SS | Oulastrea_crispata_104027 | sodium/mannose cotransporter SLC5A10-like |
| 126424:102 | pH | Oulastrea_crispata_111224 | 3-oxoacyl-[acyl-carrier-protein] reductase FabG-like |
| 24364:102 | DO | Oulastrea_crispata_9469 | kinesin-like protein unc-104 |
| 95120:79 | SS | Oulastrea_crispata_53108 | ankyrin repeat and fibronectin type-III domain-containing protein 1-like |
| 22168:21 | SS | Oulastrea_crispata_90305 | nuclear pore complex protein Nup54-like |
| 90388:71 | DO | Oulastrea_crispata_122512 | b(0,+)-type amino acid transporter 1-like |
| 89050:15 | DO | Oulastrea_crispata_121601 | fibroblast growth factor 2-like |
| 141413:114 | pH | Oulastrea_crispata_87808 | MAP kinase-activated protein kinase 5-like |
| 90388:71 | DO | Oulastrea_crispata_122513 | b(0,+)-type amino acid transporter 1-like |
| 177720:27 | TEMP | Oulastrea_crispata_6194 | protein TTE1956-like |
| 120325:113 | pH | Oulastrea_crispata_31899 | heat shock 70 kDa protein 12A-like |
| 80751:102 | pH | Oulastrea_crispata_22928 | Focal adhesion kinase 1 |
| 172491:51 | SS | Oulastrea_crispata_96972 | pleckstrin homology domain-containing family G member 3-like isoform X1 |
| 110336:88 | TEMP | Oulastrea_crispata_9341 | activating signal cointegrator 1 complex subunit 3-like |
| 123239:83 | pH | Oulastrea_crispata_103906 | lanC-like protein 3 |
| 18183:105 | SS | Oulastrea_crispata_104026 | sodium/mannose cotransporter SLC5A10-like |
| 118377:86 | DO | Oulastrea_crispata_11449 | protein-lysine N-methyltransferase EEF2KMT-like isoform X1 |
| 172491:51 | SS | Oulastrea_crispata_96963 | pleckstrin homology domain-containing family G member 3-like isoform X1 |
| 55363:84 | SS | Oulastrea_crispata_32737 | biological adhesion |

Figure S1: Pipeline for *Oulastrea crispata* RADseq processing and population genomics filtering and analyses.


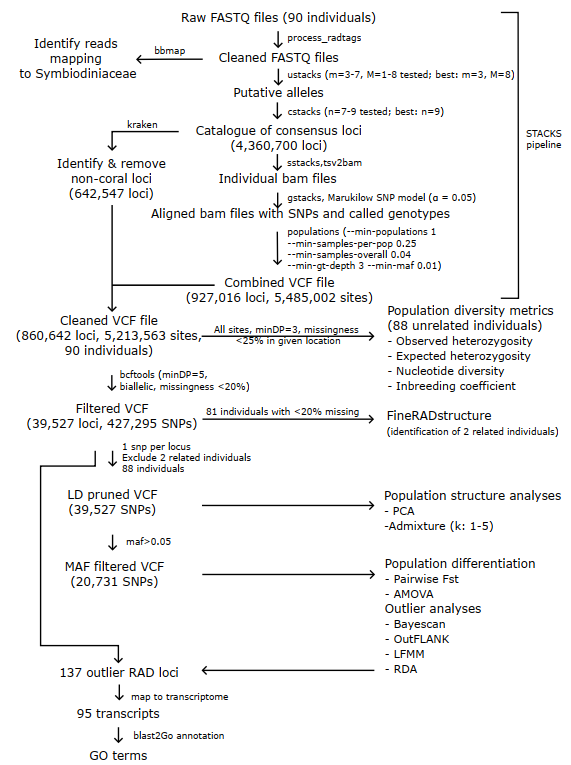


Figure S2: Principal component analysis (PCA) plots based on 39,527 SNPs from 90 individuals of *Oulastrea crispata* collected across Hong Kong. Each point is coloured according to their individual missingness across the SNP dataset.


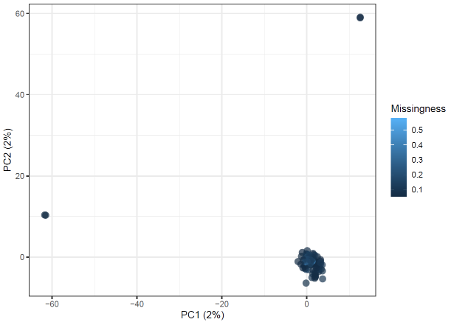


Figure S3: Pairwise co-ancestry plot based on 81 individuals of *O. crispata*, excluding individuals with more than 20% missing sites. Darker colours indicate high levels of relatedness between individuals


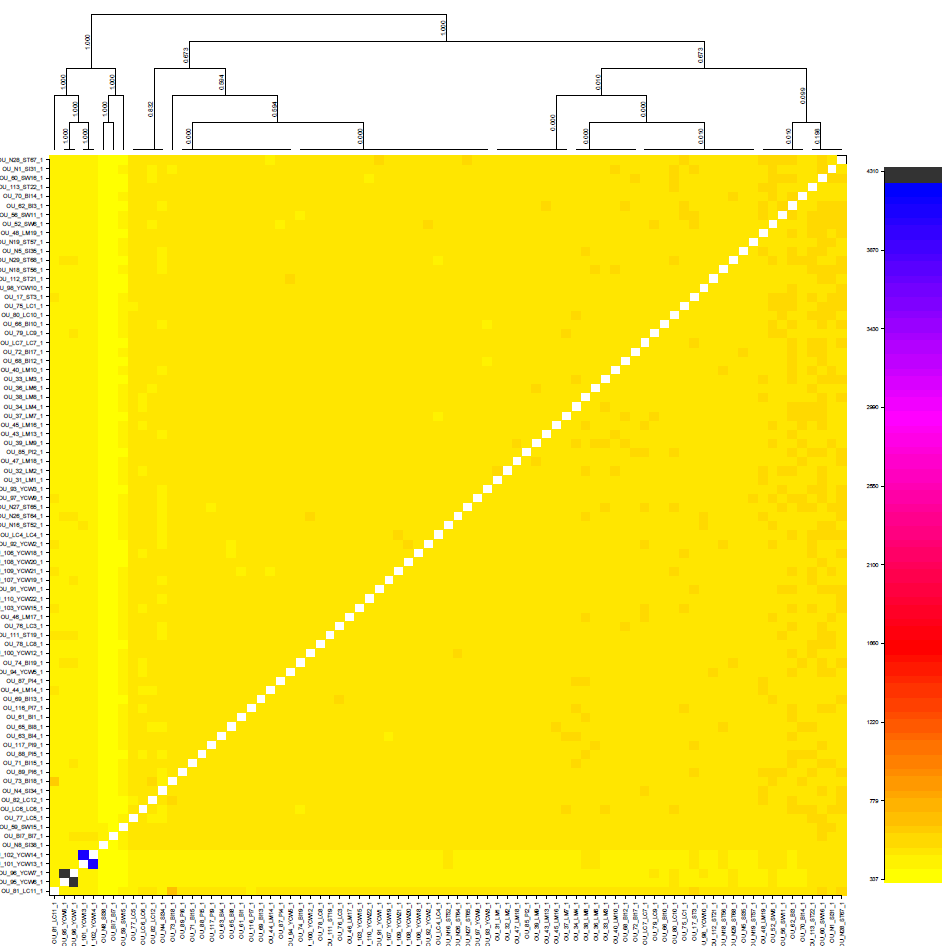


Figure S4: Admixture chromplot of K=2-5 for 90 *Oulastrea crispata* individuals grouped according to their sampling location. Colours of each bar indicate assignment into an ancestral population.


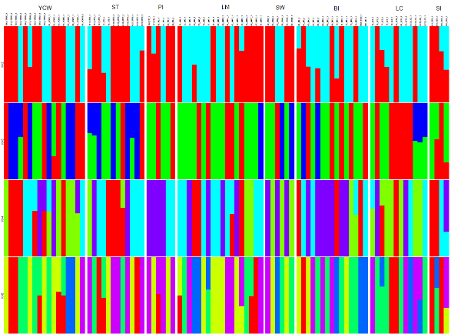


Figure S5: Redundancy analysis (RDA) bi-plot for the MAF filtered dataset of 20,731 SNPs, where grey circles indicate SNPs, coloured circles indicate individuals coloured according to their grouping (Top: water quality categories, Below: sampling location) and the arrows indicate the environmental parameters included after VIF filtering: SS: Suspended Solids, CHL: Chlorphophyll a,  DO: Dissolved oxygen, TEMP: Temperature, and pH

. 
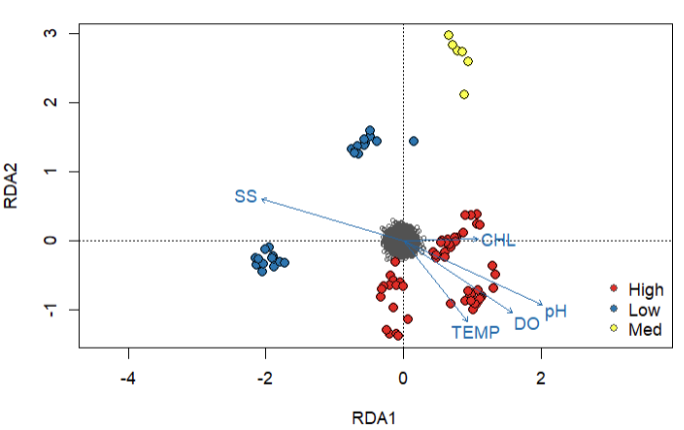

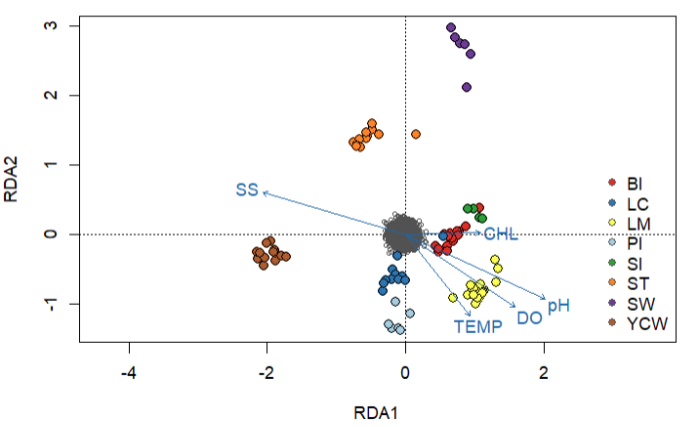


Fig S6: Identification of 137 outlier SNPs using the RDA genome-scan approach from Capblancq et al. (2018). The SNPs highlighted in orange are the outliers (q-threshold = 0.05) that are shared between the first two RDA axes that represent 43% of constrained variance attributed to environmental predictors in the dataset. Top: The plot above shows the outlier loci in a Manhattan plot, where the loci have been arranged in numerical order. Below: The outlier SNPs are highlighted in orange in the RDA bi-plot.


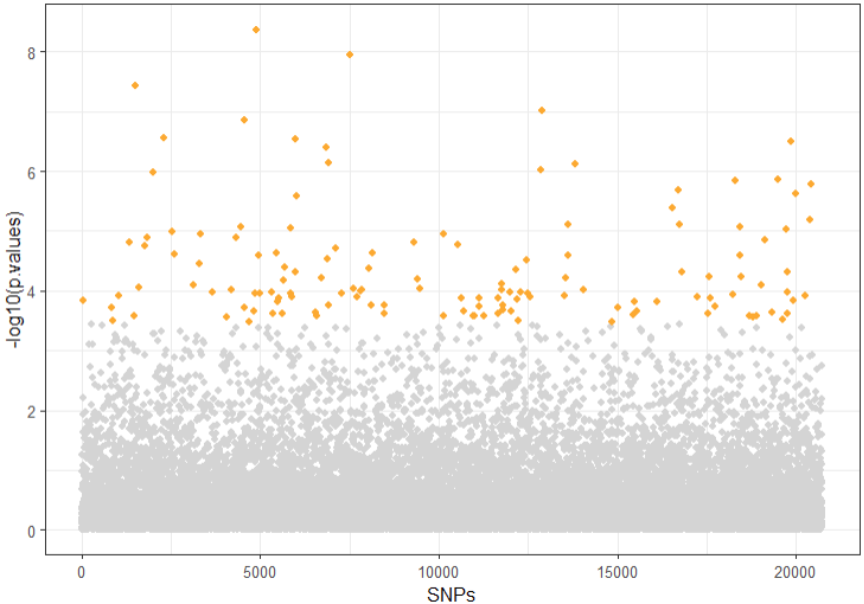


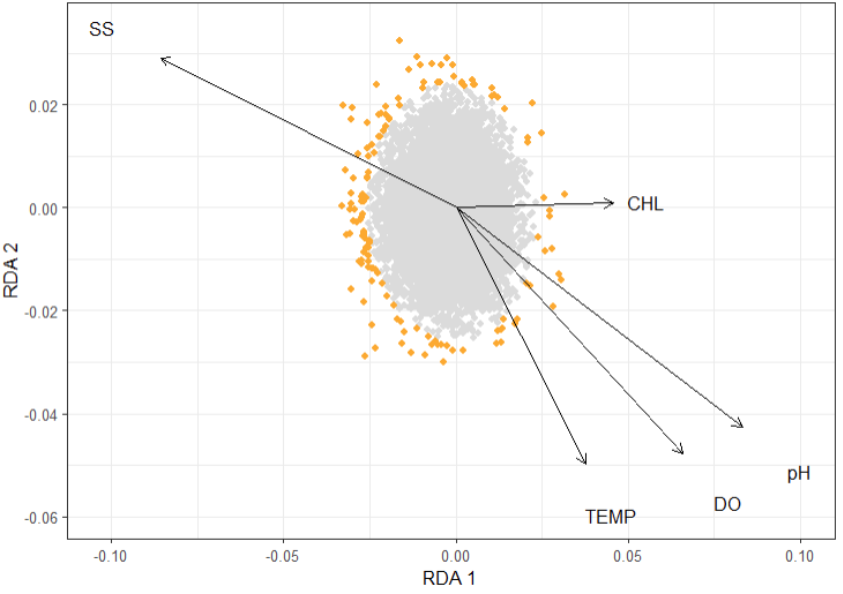


Figure S7: FST distributions for the identified outlier loci (n=137) relative to the remaining loci (n = 20594) for A) Water quality and B) Sampling locations. Higher FST differentiation is observed in the identified outlier SNPs as compared to the remaining SNPs when grouped by water quality (Welch’s t-test: t = 16.455, df = 136.58, p-value < 2.2e-16) or sampling locations (Welch’s t-test: t = 20.22, df = 137.52, p-value < 2.2e-16).


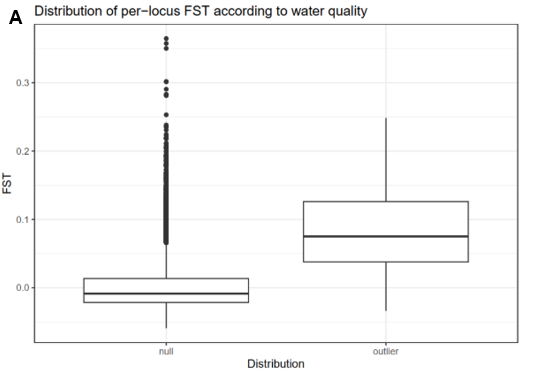

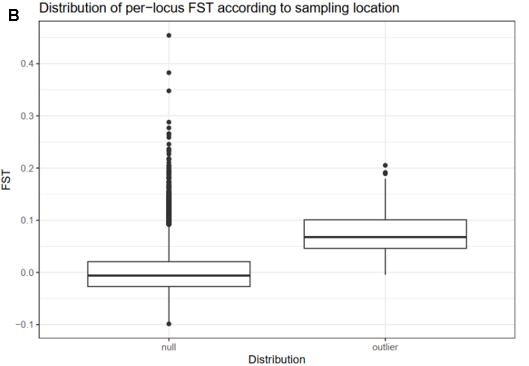


Figure S8: Boxplots of the percentage (above) and log(10) transformed percentage (below) of unambiguously mapped RADseq readsmapped to each Symbiodinaeceae reference genome, across the water quality gradient (Low, Medium and High). Note the log-scaled y-axis (below) for better visualisation as reads mapped are low across all Symbiodinaceae except for *Durusdinium trenchii*.


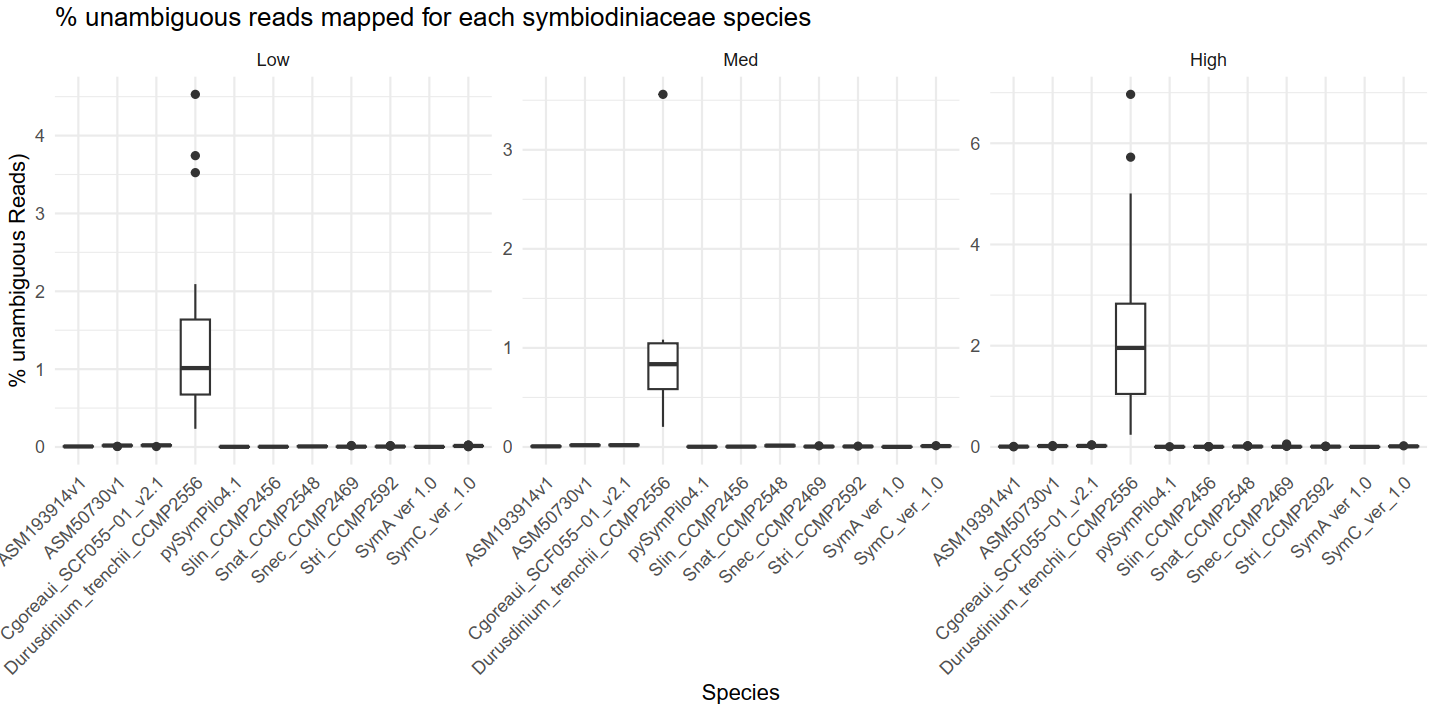


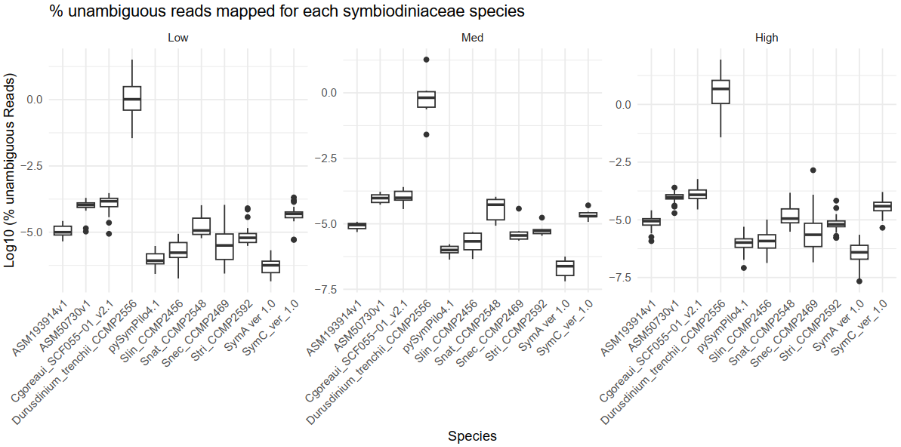

Supplement: Supplementary file 1 — Table S1: List of assembled genomes of the class Dinophyceae included for taxonomic classification of catalogue loci. Table S2: K‐values and cross‐validation (CV) error (1–5) for the admixture analysis. Table S3: Number of variant and fixed loci per sampling location used in population diversity calculations. Table S4: RDA outliers, associated loadings and correlated environmental variables. Table S5: List and frequency of GO terms identified from outlier loci. Table S6: Outlier RADseq locus and SNP from RDA analysis, associated environmental variable, best‐hit transcript identified and description of transcript from Blast2GO. Figure S1: Pipeline for Oulastrea crispata RADseq processing and population genomics filtering and analyses. Figure S2: Principal component analysis (PCA) plots based on 39,527 SNPs from 90 individuals of Oulastrea crispata collected across Hong Kong. Each point is coloured according to their individual missingness across the SNP dataset. Figure S3: Pairwise co‐ancestry plot based on 81 individuals of O. crispata , excluding individuals with more than 20% missing sites. Darker colours indicate high levels of relatedness between individuals. Figure S4: Admixture chromplot of K = 2–5 for 90 Oulastrea crispata individuals grouped according to their sampling location. Colours of each bar indicate assignment into an ancestral population. Figure S5: Redundancy analysis (RDA) bi‐plot for the MAF filtered dataset of 20,731 SNPs, where grey circles indicate SNPs, coloured circles indicate individuals coloured according to their grouping (Top: water quality categories, Below: sampling location) and the arrows indicate the environmental parameters included after VIF filtering: CHL, chlorphophyll a, DO, dissolved oxygen; SS, suspended solids; TEMP, temperature, and pH. Figure S6: Identification of 137 outlier SNPs using the RDA genome‐scan approach from Capblancq et al. (2018). The SNPs highlighted in orange are the outliers (q‐threshold = 0.05) that are [file ECE3-16-e73908-s002.docx]
